# Characteristics of late presentation of HIV infection in MSM and heterosexual adults in Portugal 2011–2013

**DOI:** 10.7448/IAS.17.4.19690

**Published:** 2014-11-02

**Authors:** Tara Shivaji, Antonio Diniz, Helena Cortes-Martins

**Affiliations:** 1 European Programme for Interventional Epidemiology Training (EPIET), Directorate General of Health, Lisbon, Portugal; 2 HIV/AIDS Programme, Directorate General of Health, Lisbon, Portugal; 3 HIV/AIDS Surveillance, Instituto Nacional de Saúde, Doutor Ricardo Jorge, Lisbon, Portugal

## Abstract

**Introduction:**

It is estimated that over half of newly diagnosed HIV infections in Europe present late. An HIV positive individual presenting late to care represents a missed opportunity to benefit from treatment, increasing the risk of morbidity and mortality at an individual level, and onward disease transmission at population level. Reducing late presentation is a strategic priority of the Portuguese HIV/AIDS program. We set out to describe the characteristics of late presentation in the two largest transmission risk groups currently in Portugal to inform HIV prevention and treatment.

**Methods:**

We extracted details of all notified cases from individuals over 15 years with a laboratory confirmed HIV diagnosis made between January 2011 and December 2013 from the Portuguese HIV surveillance database and selected cases from heterosexuals and men who have sex with men (MSM). We defined late presentation as an initial CD4 count <350 cells/mm^3^ or presence of AIDS-defining disease at time of HIV diagnosis. We calculated adjusted odds ratios (aOR) with 95% confidence intervals (CI) for characteristics associated with late presentation in separate logistic regression for heterosexuals and MSM.

**Results:**

Of 4219 HIV positive cases notified, 2589 (61%) were heterosexuals and 1220 (29%) were MSM. A total of 1586 (38%) cases presented late of which 1037 (40%) were heterosexual and 328 (27%) were MSM. The median age at late presentation was 40 in heterosexual women, 46 in heterosexual men and 35 in MSM. A total of 1446 (55%) of heterosexual HIV positive adults were unaware of having had a high risk sexual contact. Late presentation among heterosexuals was associated with being male (aOR=1.58 95% CI 1.29–1.93), not knowing a partners’ HIV status (OR=1.59 95% CI 1.35–1.87) and age, increasing the odds of late presentation by a factor of 1.02 per year (95% CI 1.01–1.03). Among MSM, only age was associated with late presentation, increasing by 1.04 (95% CI 1.03–1.05) per year.

**Conclusions:**

Portuguese HIV prevention programs need to increase the risk awareness of heterosexuals, particularly men, to reduce missed opportunities for early diagnosis. As half of all cases presenting late are aged 40 and over, we recommend that general HIV outreach services and specialist services for MSM review their accessibility and acceptability to both middle and older-aged clients.

